# Supplementary figures and images for: Identification of Candidate Genes for Red-Eyed (Albinism) Domestic Guppies Using Genomic and Transcriptomic Analyses
Source: Int J Mol Sci. 2024 Feb 11;25(4):2175. doi: 10.3390/ijms25042175 (PMC10888696; doi:10.3390/ijms25042175)

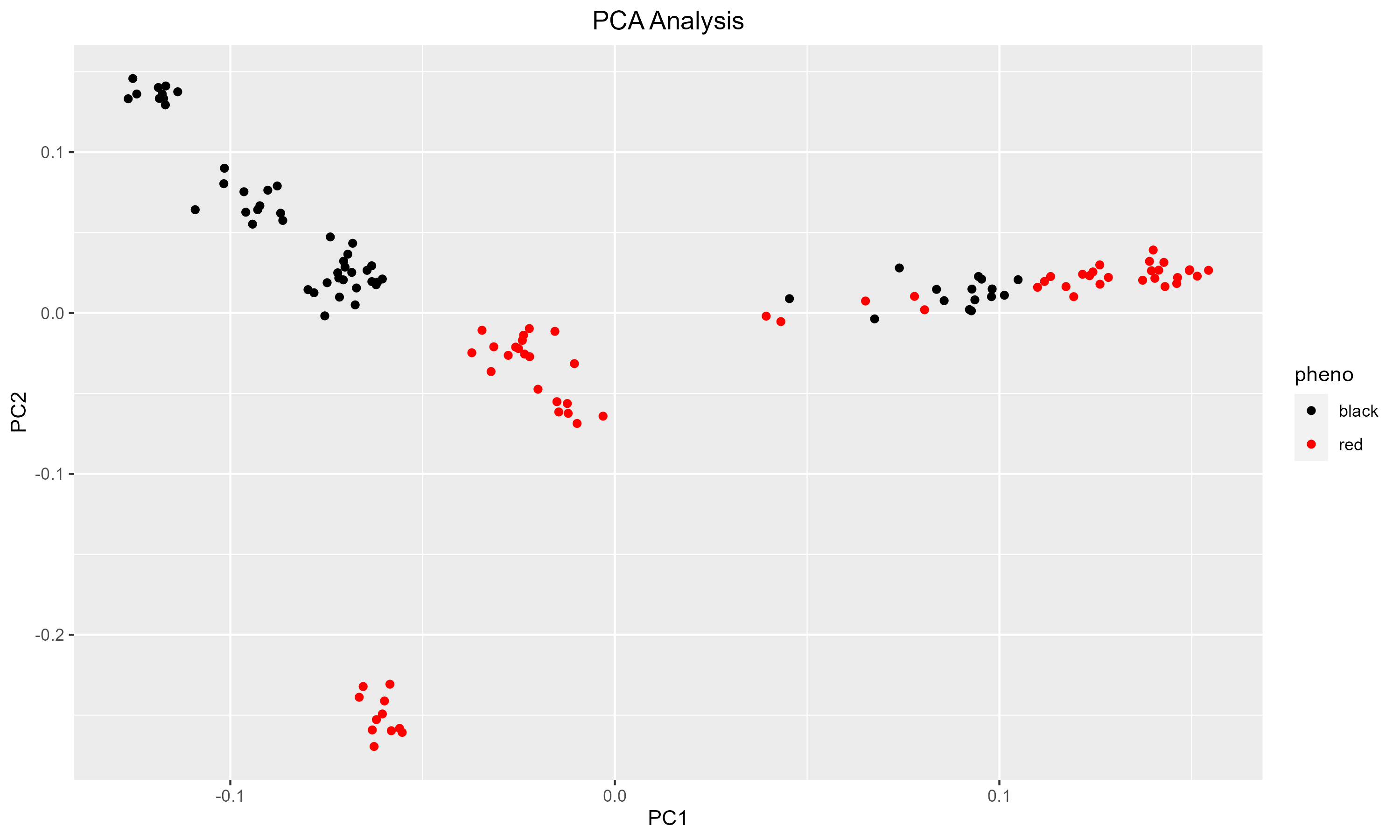

Supplement: Supplementary file 1 [file ijms-25-02175-s001.zip › Figure S1 The plot of PCA analysis.tiff]
